# Adverse drug events in hospitalized children at Ethiopian University Hospital: a prospective observational study

**DOI:** 10.1186/s12887-015-0401-0

**Published:** 2015-07-15

**Authors:** Tesfahun Chanie Eshetie, Bisrat Hailemeskel, Negussu Mekonnen, Getahun Paulos, Alemayehu Berhane Mekonnen, Tsinuel Girma

**Affiliations:** School of Pharmacy, College of Public Health and Medical Sciences, Jimma University, Jimma, Ethiopia; College of Pharmacy, Howard University, 2300 4th Street, N.W, Washington, DC 20059 USA; Management Sciences for Health - Ethiopia, P.O Box: 1157, Code 1250 Addis Ababa, Ethiopia; School of Pharmacy, Clinical Pharmacy Unit, University of Gondar, P.O Box: 196, Gondar, Ethiopia; Department of Pediatrics and Child Health, College of Public Health and Medical Sciences, Jimma University, Jimma, Ethiopia

**Keywords:** Adverse drug events, Ethiopia, Hospitalized children, Incidence, Risk factors

## Abstract

**Background:**

The nature and magnitude of adverse drug events (ADEs) among hospitalized children in low-income countries is not well described. The aim of this study was thus, to assess the incidence and nature of ADEs in hospitalized children at a teaching hospital in Ethiopia.

**Methods:**

We used prospective observational method to study children that were hospitalized to Jimma University Specialized Hospital between 1 February and 1 May 2011. ADEs were identified using review of treatment charts, interview of patient and care-giver, attendance at ward rounds and/or meetings and voluntary staff reports. Two senior pediatric residents evaluated the severity and preventability of ADEs using preset criteria. Logistic regression analysis was employed to determine predictors of ADEs.

**Results:**

There were 634 admissions with 6182 patient-days of hospital stay. There were 2072 written medication orders accounting for 35,117 medication doses. Fifty eight ADEs were identified with an incidence of 9.2 per 100 admissions, 1.7 per 1000 medication doses and 9.4 per 1000 patient-days. One-third of ADEs were preventable; 47 % of these were due to errors in the administration stage of medication use process. Regarding the severity of ADEs, 91 % caused temporary harms and 9 % resulted in permanent harm/death. Anti-infective drugs were the most common medications associated with ADEs. The occurrence of ADEs increased with age, length of hospital stay, and use of CNS, endocrine and antihistamine medicines.

**Conclusion:**

ADEs are common in hospitalized children in low-income settings; however, one-third deemed preventable. A strategy to prevent the occurrence and consequences of ADEs including education of nurses/physicians is of paramount importance.

**Electronic supplementary material:**

The online version of this article (doi:10.1186/s12887-015-0401-0) contains supplementary material, which is available to authorized users.

## Background

Adverse events, injuries that occur during medical management, have received attention after the Harvard Medical Practice Study [1, 2]. Brennan et al. [1] estimated that 3.7 % of all hospitalized patients experienced an adverse event. In 1999, the Institute of Medicine of USA [3] reported that preventable medication related events alone could result in 7000 deaths annually.

Despite the extensive literature on adverse events due to medications (also known as adverse drug events) in adult populations, there is scarcity of published study on pediatric-specific adverse drug events (ADEs) [4, 5]. But, there are suggestions that harm to medication use might be higher in children than in adults [6]. The rate of pediatric ADEs rates can range from 6.6 to 15.7 events per 1000 patient-days [4, 7] and 1.2 per 1000 medication doses [7], with a potential ADE rate of 10 per 100 admissions [8]. However, studies that utilized adverse drug reaction (ADR) as an outcome reported a higher incidence of ADRs in pediatric inpatients, 10 to 17 % [9–14]. Moreover, in another meta-analysis of prospective studies the overall incidence of ADRs was found to be 9.5 %; severe reactions accounted for 12.3 % of the total [15].

The incidences of adverse drug events may vary depend on study definitions and detection methods. The available methods of detection include voluntary reporting [16–18], patient interviews and chart reviews [16], trigger tools [19] and computerized monitoring systems [20–23]. While spontaneous reporting underestimates the real incidence of ADEs [24], the use of computerized monitoring system generates the best results [22, 23]. Since there is no single best method [20], the use of multiple strategies maximizes the incidence of ADE quantification [23].

ADEs are a major burden on healthcare; the consequences can vary from temporary/permanent harms [25] to costs associated with ADE management [3, 9, 26–28] and prolonged hospital stay [29, 30]. Studies have shown that 2 % of hospital admissions are due to ADEs in the pediatrics [12, 31], but a higher admission is reported in patients with off-label use of medications, 11 % [32]. A Canadian study in the pediatric patients described that 8 % of emergency department visits were attributed to medication-related events, of which two-third were deemed preventable [33]. However, there is paucity of information with regard to the magnitude of ADEs in hospitalized children in developing countries including Africa. Thus, the aim of this study was to assess the incidence and nature of ADEs in hospitalized children in the pediatric ward of a teaching hospital in Ethiopia.

## Methods

### Study setting and design

This prospective observational study was conducted in the pediatric ward of Jimma University Specialized Hospital (JUSH), Ethiopia, which had a bed capacity of 503. The hospital was providing both outpatient and inpatient pediatric services for children less than 14 years. During the study period, the inpatient department had four units i.e., critical care, neonatal (for neonates ≤ 14 days of age), nutritional rehabilitation and general. There were 4 pediatricians, 5 senior residents, 12 junior residents and 20 nurses during the study period.

All pediatric inpatients that were hospitalized between 1 February and 1 May 2011 were included. Patients were excluded if the hospital admission was for less than 24 h, and/or if the admission was the result of an intentional (self-administered) overdose. All admitted pediatric patients were followed for the main outcome measure (occurrence of actual ADEs) from admission to discharge/transfer/death. In addition, the preventability and severity of each ADE episode was evaluated.

### Data collection and ADE case evaluation

ADE was defined as any incident resulting in injury from any stage of the medication use process (ordering, transcribing, dispensing, administrating and monitoring) [34]. A preventable ADE was an injury due to an error at any stage in the medication use - for instance, hypoglycemia due to insulin overdose. Non-preventable ADE was an injury not related to error in the medication process. An allergic reaction in a patient not previously known to be allergic to the medication is an example of non-preventable ADE.

A combination of methods was employed to identify ADEs. From the patient medical record, one nurse & two pharmacists collected the following demographic and clinical data from medical records of participants using a structured format: age, gender, history of previous medication and drug allergy, admission diagnoses, current drug dosage and regimen and length of hospital stay. Charts were reviewed daily until discharge/transfer/death of the child. Moreover, changes in medication regimens including discontinuation or initiation of new medications and abnormal laboratory values were recorded. A list of pediatric trigger tools, these are drugs or clues that have links to potential ADEs because either they are antidotes or given to reverse the action of a drug responsible for ADE, was adapted from US organizations [35, 36] and modified based on availability of medicines in Ethiopia [37] (Additional file 1).

In case of medication management changes, the responsible physician was contacted for clarification. Evaluation was made whether the change was due to ADE. Besides, we asked the pediatric ward staff to report any actual events or potentially unsafe medication systems that was noticed. A clinical pharmacist was attending clinical rounds/meetings and visited the ward daily to solicit any alerts for ADE. The clinical pharmacist forwarded any suspected ADE cases for further evaluation to a multidisciplinary team comprised of senior attending physician, pediatric resident, nurses and pharmacists. Once decided by the multidisciplinary, the suspected ADE was assessed for temporal relationship between the drug and the event as per WHO-UMC criteria [38].

The response to withdrawal plausibility, if possible was also evaluated. Those in the category of possible, probable/likely and certain were considered. We searched biomedical literatures to establish the strength of published data, if any, on the relationship between the ADEs and the medication. During this evaluation, the expertise of the pediatrics team was used when required for further work-up especially on the exclusion of possible disease condition. Since ADEs were actual patient harms, a specific medical care was given when applicable to prevent further damage.

For categorizing severity of ADEs, the National Coordinating Council for Medication Error Reporting and Prevention (NCC MERP) scale was employed [39]. Each event was assigned a harm level of E - I. Severity category E, F, G, H and I referred to temporary harm to the patient requiring intervention, temporary harm to the patient requiring initial or prolonged hospitalization, permanent harm to the patient, intervention required to sustain life, e.g. cardiovascular/respiratory support and death of the patient respectively.

Preventability was determined using the explicit criteria developed by Schmumock and Thornton [40]. Categorization of events (severity and preventability) was evaluated by a panel of two senior pediatric residents, who independently classified the events using preset criteria. The reviewers reached consensus through discussion for discordant classification.

### Data analysis

Data were analyzed using Statistical Package for the Social Sciences (SPSS Inc., Chicago, IL, USA) version 16. Qualitative variables were described as frequencies (percentages) and quantitative variables as mean ± standard deviation (SD). Each ADE was treated in the analysis as a separate independent ADE. ADE incidence was calculated per 100 admissions, per 1000 patient-days, per 1000 medication doses and per 100 medication orders. Kappa statistics were used to determine inter-rater reliabilities. Covariates for occurrence of ADEs were evaluated using logistic regression analysis. The covariates were number of medications ordered, length of hospital stay, age, medication class and presence of infectious disease. Odds ratio (crude and adjusted) with its *p*-value and 95 % confidence interval was reported. A *p*-value <0.05 was considered statistically significant.

### Ethical consideration

Ethical approval was obtained from Ethical Review Board of Jimma University. Patient/caregiver/family member gave verbal consent for the information required. Appropriate interventions were recommended to the pediatrics team when serious medical errors were identified.

## Results

### Study population characteristics

A total of 699 admitted patients were followed. Among these, 65 admissions were excluded (length of stay less than 24 h and/or insufficient data). We included 634 admissions representing 600 patients for analysis.

**Fig. 1:**
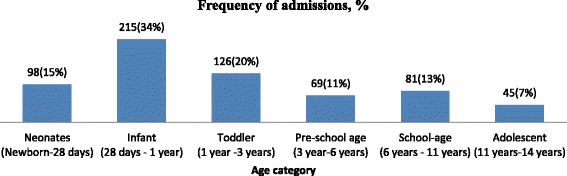
The age category of hospitalized children in Jimma University Specialized Hospital

Pediatrics of various ages were included (minimum 7 days, maximum 14 years). The mean age of the patients was 2.9 years, 371 (61.8 %) were males and 215 (33.9 %) infants (Fig. 1). The length of hospital stay was 6182 patient-days. A total of 2072 medication orders were written accounting for 35,117 medication doses and 55.4 medication doses per admission. Of those included in the analysis, 15 (2.5 %) admissions didn’t receive any medication during their hospital stay. The mean ± SD length of hospital stay and medications ordered were 9.8 ± 8.8 days and 3.3 ± 1.9 respectively.

The top 10 admitting diagnosis were severe pneumonia 173 (27.3 %), severe acute malnutrition 120 (18.9 %), early/late onset neonatal sepsis 108 (17.0 %), meningitis 59 (9.3 %), acute gastroenteritis 46 (7.3 %), malaria 39 (6.2 %), anemia’s of different causes 43 (6.8 %), first episode of wheeze 32 (5.1 %), congestive heart failure 27 (4.3 %) and soft tissues abscess 26 (4.1 %). Anti-infective drugs, 1330 (64.2 %) were the leading class of medications prescribed followed by drugs acting on the central nervous system (CNS), 206 (9.9 %) (Table 1).

**Table 1:** Frequency of medication classes prescribed for hospitalized children in Jimma University Specialized Hospital

Code^b^	Medication class	Frequency of prescription (*N* = 2072), %
AI.000	Anti-infective medicines	1330 (64.2)
NS.000	Central nervous system medicines	206 (9.9)
VT.000	Vitamins	158 (7.6)
CV.000	Cardiovascular medicines	103 (5.0)
RE.000	Respiratory medicines	66 (3.3)
ED.000	Medicines used in endocrine disorders	66 (3.3)
OP.000	Ophthalmic agents	30 (1.5)
BL.000	Blood products and medicines affecting the blood	28 (1.4)
DE.000	Dermatological agents	25 (1.2)
GI.000	Gastrointestinal medicines	20 (1.0)
AL.000	Antihistamines and anti-allergic medicines	10 (0.5)
MS.000	Medicines used in musculoskeletal and joint diseases	5 (0.2)
--------	Others^a^	25 (1.2)

^a^Other includes calcium gluconate, calvitalis® (consisted of calcium and other 9 vitamins), magnesium sulfate, etc

^b^Code given is based on Pharmacologic – Therapeutic classification scheme used in the list of medicines in Ethiopia, 2010 [37]; this schematic classification is analogous to ATC codes employed elsewhere

### Incidence, preventability and severity of ADEs

A total of 58 ADEs were identified in 46 patients. The incidences of ADEs were found to be 9.2 per 100 admissions (crude rate), 1.7 per 1000 medication doses, 9.4 per 1000 patient days and 2.8 per 100 medication orders. The majority of ADEs occurred in the general pediatric ward, 33 (56.9 %) followed by the critical unit, 21 (36.2 %). Twelve patients had more than 1 ADEs during hospitalization. 4 of the 58 (6.9 %) ADEs were the primary reasons for hospitalization i.e. these ADEs were a cause of hospital admissions. For example, a child with known type I diabetes was admitted as result of severe hypoglycemia following insulin injection.

Thirteen (22.4 %) of the ADEs were injection site phlebitis (pain, swelling and redness) and 12 (20.7 %) were maculopapular skin rash with or without urticaria (Table 2). Of the 58 ADEs, the reviewers classified 39 (67.2 %) as non-preventable and 19 (32.8 %) as preventable. Among the preventable ADEs, 9 (47 %) were following errors in the administration stage of medication use process, 8 (42 %) due to improper dosage and 2 (11 %) were attributed to monitoring errors. Examples of preventable ADEs are listed in Table 3.

**Table 2:** Types of ADEs identified clinically among children admitted in Jimma University Specialized Hospital

Adverse drug event	n (%)
Injection site phlebitis	13 (22.4)
Skin rash with/without urticaria	12 (20.7)
N/V, dyspepsia (+/− loss of appetite)^a^	7 (12)
Antibiotic associated diarrhea	6 (10.3)
Infiltration, subcutaneous	3 (5.2)
Oral candidiasis	2 (3.4)
Extravasation induced tissue necrosis	2 (3.4)
Others^b^	16 (27.6)

^a^N/V (nausea or vomiting), +/− (with or without)

^b^Includes hypotension, tachycardia, pain/burning sensation at injection site, hypoglycemia, congestive heart failure (aggravated), acute dystonic reaction, rectal irritation (proctitis), gangrene, gingival hypertrophy/facial coarsening, DKA/hyperglycemia, over sedation, irritability, exoflative dermatitis, seizure, headache/abdominal pain and death

**Table 3:** Examples of preventable ADEs that occurred at different stages of the medication use process in children admitted in Jimma University Specialized Hospital

Stage of error	Description of case
Administration	A child admitted with newly diagnosed type I DM without DKA developed moderate DKA while in the hospital due to omissions of insulin dose
	For a newborn, a nurse secured IV line and inadvertent intra-arterial administration of Ampicillin and Gentamicin lead to extravasation induced necrosis
	A child with pharyngitis who was taking IV cloxacillin developed infiltration at the injection site
Prescribing	An 8 year old severely malnourished child with the diagnosis of CHF secondary to chronic valvular heart disease was receiving Lasix 20 mg PO BID, and digoxin 0.125 mg P.O per day, who latter developed irritability
	Over sedation due to an overdose of tramadol in a child with moderate pain
	A child developed maculopapular rash with urticaria to cloxacillin with previous history of penicillin allergy
Monitoring	A 6 month infant with severe pneumonia was put on crystalline penicillin but failure to use appropriate clinical or laboratory data for adequate assessment of patient response to prescribed therapy cause the death of a patient

*BID* (*bis in die*) twice daily, *CHF* congestive heart failure, *DM* diabetes mellitus, *DKA* diabetic ketoacidosis, *PO* per oral

Majority of the ADEs were of temporary harms i.e. 39 (67 %) category E, and 14 (24 %) category F. Only 5 (9 %) of the ADEs resulted in permanent harm/death. Three out of four permanent harms were due to wrong administration of drugs. There was one death associated with monitoring error; it was most likely due to a failure to use appropriate clinical or laboratory data to monitor response to crystalline penicillin prescribed for severe pneumonia. The level of agreement between reviewers for severity rating of ADE was “good” (Kappa = 0.65) whereas it was “moderate” (Kappa = 0.46) for preventability.

Anti-infective was the most common medication class responsible for the ADEs (Table 4). Injection site phlebitis, skin rash with/without urticaria and antibiotic associated colitis were the common ADEs associated with anti-infectives. Of 58 ADEs, 21 (36.2 %) were detected in infants and 14 (24.1 %) in school-age children. Thirty nine (67.2 %) of all the ADEs occurred with IV route of administration and 16 (27.6 %) followed oral route. Drugs were used to manage 30 (51.7 %) ADEs while the offending drugs were discontinued in 16 (27.6 %) cases. Fifteen (25.8 %) ADEs required increased monitoring of vital signs and/or laboratory values (e.g. serial of random blood glucose level determinations) and 4 (6.9 %) ADEs required dose reduction. Other interventions included change of the IV injection site, saline flushing of the IV line, daily wound care and abscess drainage.

**Table 4:** The classes of medications responsible for adverse drug events among children hospitalized in Jimma University Specialized Hospital

Medication class^a^	n (%)
Anti-infective medicines^b^	42 (72)
Cardiovascular medicines	4 (7)
Central nervous system medicines	4 (7)
Respiratory medicines	3 (5)
Medicines used in endocrine disorders	3 (5)
Gastrointestinal medicines	1 (2)
Medicines affecting the blood	1 (2)

^a^Classification is based on Pharmacologic – Therapeutic classification scheme used in the list of medicines in Ethiopia, Sept 2010 [37]

^b^For one ADE, the maintenance fluid (isotonic normal saline) also contributed for infiltration in additions to anti-infectives being used

In the multivariable analysis, length of stay greater than 23 days (AOR 8, 95 % CI: 2.93, 22.04), presence of infectious disease (AOR 3.43, 95 % CI: 1.19, 9.91), use of anti-histamines and anti-allergic (AOR 32.5, 95 % CI: 6.0, 176.45), CNS (AOR 2.09, 95 % CI: 1.01, 4.32) and endocrine (AOR 3.38, 95 % CI: 1.40, 8.15) medicines were associated with occurrence of ADEs (Table 5).

**Table 5:** Odds ratio for factors associated with ADEs among children hospitalized in Jimma University Specialized Hospital

Characteristics	ADEs occurred		Crude OR (95 % CI)	Adjusted OR^b^(95 % CI)
	Yes (*n* = 46)	No (*n* = 588)		
Number of medications ordered^a^				
1–5	34	512	1.0	1.0
6–10	11	58	2.86 (1.37–5.94) **	0.76 (0.26–2.18)
≥11	1	3	5.02 (0.51–49.55)	0.17 (0.00–11.22)
Length of hospital stay				
1–8	12	352	1.0	1.0
9–15	11	132	2.56 (1.10–5.95) **	2.47 (1.00–6.14)
16–22	11	62	5.20 (2.20–12.32)*	5.06 (1.98–12.94) **
≥23	12	42	8.38 (3.54–19.84) *	8.04 (2.93–22.04) *
Age (Years)				
Neonate	4	94	1.0	1.0
Infant	16	199	1.89 (0.61–5.81)	1.29 (0.39–4.23)
Toddler	6	120	1.17 (0.32–4.28)	0.39 (0.09–1.74)
Pre-school age	1	68	0.36 (0.04–3.16)	0.18 (0.02–1.87)
School age	11	70	3.69 (1.13–12.08) **	1.94 (0.53–7.12)
Adolescent	8	37	5.08 (1.44–17.90) **	2.69 (0.67–10.74)
Use of CNS medicines				
No	24	155	1.0	1.0
Yes	22	433	2.56 (1.40–4.70) **	2.09 (1.01–4.32) **
Use of endocrine medicines				
No	35	537	1.0	1.0
Yes	11	51	3.31 (1.58–6.91) **	3.38 (1.40–8.15) **
Use of other medicines				
No	42	572	1.0	1.0
Yes	4	16	3.40 (1.09–10.64) **	1.79 (0.37–8.65)
Use of anti-histamine and anti-allergic				
No	40	584	1.0	1.0
Yes	6	4	21.90 (5.94–80.80)*	32.51 (5.99–176.45)*
Presence of infectious disease				
No	5	185	1.0	1.0
Yes	41	403	3.76 (1.46–9.68) **	3.43 (1.19–9.91) **

* *p* < 0.001, **p < 0.05

^a^15 patient admissions were not taking any medications. *N* = 619, ‘Yes’ = 46; ‘No’ = 573

^b^The odds ratio was adjusted for number of medications, length of hospital stay, age (years), use of CNS medicines, use of endocrine medicines, use of other medicines, use of antihistamine and anti-allergic and presence of infectious disease

## Discussion

In countries with better resources, medication safety programs are well integrated with the health care system [7, 9, 10]. In low income countries like Ethiopia, however, healthcare coverage is prioritized to medication safety. Moreover, the medication use system is not evidence based. To our knowledge, this is the first study from Ethiopia assessing the extent of ADEs in children and hence, this study will provide baseline data for patient safety advocates.

Estimation of the incidence of ADEs significantly depends on the trigger to which the event was searched, the methodology and definition used. The incidence rates of ADEs in our study were higher when compared with two studies in the USA; 6 per 100 admissions and 7.5 per 1000 patient-days [41] and 2.3 per 100 admissions and 6.6 per 1000 patient-days [4]. But the rates were lower than a finding from New Zealand study (12.9 per 100 admissions and 22.1 per 1000 patient-days) [9]. Another study from USA [7], conducted through a retrospective focused chart review and pediatric trigger tools, reported an ADEs incidence of 11.1 per 100 admissions, 15.7 per 1000 patient-days and 1.23 per 1000 medication doses. A similar study definition was employed in Takata et al. study [7], but a little bit higher ADE incidence was reported. It shouldn’t be mistakenly noticed that our finding is lower than that of the USA because of the methodology used is different (retrospective, focused chart review). But, when we compared with Kaushal et al. [4] and Holdsworth et al. [41] studies that used similar methods, we found a higher rate of ADEs.

Comparing the preventability of ADEs in our study with previous studies, different authors reported different figures: Holdsworth et al. [41] 61 %, Kaushal et al. [4] 19.2 %, Kunnac et al. [9] 57 % and Takata et al. [7] 29 %. In this study, the most common medication errors responsible for preventable ADEs were errors occurred during administration and improper dose (dose too low/high). According to Takata et al. [7], most of preventable ADEs occurred during monitoring stage; defined as a failure to use appropriate clinical or laboratory data for adequate assessment of patient response to prescribed therapy.

In this study, the severity rating for observed ADEs showed that around 91 % resulted in temporary harm (either category E or G). Takata et al. [7], reported that all of the ADEs they detected caused temporary harm. Other than the NCC MERP scale, other previous studies also reported serious to life-threatening events in 24–34 % of ADEs [4, 41], and permanent harms in 5 % of ADEs [41]. Thus, comparatively the severity of ADEs in our study (9 % resulted in permanent harm/death) was very high. In this study, 3 of the 4 events that resulted in permanent harm were due to inadvertent route of administration of medication, and were classified as preventable. The incidence of ADEs was comparable with most previous reports, but we can still appreciate that hospitalized children in our setting were facing a considerable amount of preventable medication-related permanent harm.

In our study, injection site phlebitis was the commonest ADEs detected. Kaushal et al. [4] reported, two third of non-preventable ADEs were related to antibiotic associated *Clostriduim difficile* infections, rashes, allergic reactions and yeast infection. Again similar findings were reported by Takata et al. [7] where pruritus was the most common ADE.

In our study, the most common medication class responsible for the ADEs was anti-infectives. This medication class was also the most commonly used medication classes among all study participants. In other studies, the most commonly cited medication classes associated with ADEs were analgesics/opioids followed by antibiotics [7, 41]. Opioids are mentioned frequently as a cause of ADEs in the literature but they were not available in JUSH during the study period.

Additional interventions were required to manage ADEs. ADE management was mostly done through prescriptions of additional medications followed by discontinuation of the offending agent. These additional interventions can predict the impact of ADE on this hospital as well as to the patient. In developing countries like Ethiopia, these interventions are associated with immense cost imposition to the health system.

Presence of infectious disease, use of antihistamines and anti-allergic, CNS and endocrine medicines was associated with occurrence of ADEs. The use of anti-histamine and anti-allergic medications were strongly associated with ADEs. This interpretation should be in caution, however. None of the antihistamine and anti-allergic drugs were implicated in causing ADEs, but 6 of the 10 prescriptions were written for reversing the causality. This correlates well to the notion that during monitoring for occurrence of ADEs, the use of anti-histamines and anti-allergic medications give a clue for further evaluation of ADEs.

We found that ADEs increased with the length of hospital stay akin to a finding by Holdsworth et al. [41]. Santos et al. [13] have also reported that children with longer length of stay, greater number of medications had higher ADR incidence. In one study among adults [42], the authors identified that exposure to psychoactive and CV drugs were independent correlates of preventable ADEs. This was similar to our findings which showed CNS and endocrine medicines and presence of infectious disease as strong predictors of ADE.

This study has limitations, however. It is a single center study and therefore might not be generalized to other hospitals in Ethiopia. The incidence of ADEs might have been underestimated as some ADES may not have been recorded in the charts and may thus have not been detected. Any event that has occurred in patients with less than 24 h of hospital stay was not included but it was unlikely that we missed those events as such events required prolonged stay.

## Conclusion

The incidence of ADE was high among children hospitalized to JUSH and ADEs were more likely to occur among children with longer length of hospital stay, presence of infectious disease, use of CNS, endocrine and anti-histamine medications. Anti-infectives were the most commonly implicated drugs for development of ADEs. Only one third of ADEs were found to be preventable. Though most of the ADEs were evaluated to cause temporary harm, clinically significant number of children suffered from permanent harm. This calls for a strategy to prevent the occurrence and consequences of ADEs in the pediatric ward of JUSH including education of nurses/physicians.

## Additional file

Additional file 1:
**Trigger tools or clues for a focused chart review.**

## Abbreviations

ADE: Adverse drug event
ADR: Adverse drug reaction
AOR: Adjusted odds ratio
CNS: Central nervous system
CV: Cardiovascular
JUSH: Jimma University Specialized Hospital
NCC MERP: National Coordinating Council for Medication Error Reporting and Prevention Scale
SD: Standard deviation
WHO-UMC: World Health Organization- Uppsala Monitoring Centre
