# Supplementary material for: Adverse drug events in hospitalized children at Ethiopian University Hospital: a prospective observational study
Source: BMC Pediatr. 2015 Jul 15;15:83. doi: 10.1186/s12887-015-0401-0 (PMC4502527; doi:10.1186/s12887-015-0401-0)
Supplement: Supplementary file 1 [file 12887_2015_401_MOESM1_ESM.docx]

Additional file

1. Trigger tools or clues for a focused chart review

A. Triggers medications

Trigger drugs are drugs that are links to a possible adverse drug events because antidotes or are given to reverse the action of a drug.

1. Atropine - bradycardia
2. Benzatropine/Trihexyphenidyl - extra pyramidal reactions
3. Blood transfusions - NSAID or drug induced gastric bleeds
4. Calcium chloride - Calcium channel blocker overdose
5. Dantrolene - hyperthermia, neuroleptic malignant syndrome
6. Dextrose 50% in water - hypoglycemia
7. Diazepam - drug induced seizures
8. Digoxin immune fab (Digibind) - digoxin overdose
9. Diphenhydramine - hypersensitivity reactions, drug rashes, extra pyram
10. Epinephrine - hypersensitivity reactions
11. Flumazenil - benzodiazepine overdose
12. Fosphenytoin/Phenytoin - seizures, arrhythmias
13. Glucagon - hypoglycemia, beta blocker overdose
14. Naloxone - narcotic overdose
15. Phentolamine - dopamine extravasation

16 Vitamin K (Phytonadione) - Warfarin toxicity or hypoprothrombinemia

B. <u>New symptoms or events as triggers that may show a possible adverse drug</u>

1. PTT > 100 seconds**:** This is not an infrequent occurrence when patients are on
   With Vitamin K, look for evidence of bleeding to determine if an ADE has oc
2. Rising serum creatinine**:** A rising serum creatinine is defined as a serum crea
   Becomes elevated relative to age-specific normal values or as an increase in s
   Creatinine of >=0.4mg/dl.
3. Over sedation, lethargy, falls, hypotension: If found, look for a relationship be
   event and administration of a sedative, analgesic, or muscle relaxant.
4. Rash: There are many causes for a rash. Look for evidence that the rash is rel
   administration, including overuse of antibiotics resulting in yeast infections.
5. Abrupt medication stop: In the order sets, whenever "hold" or "stop" medicati
   Appear, look for the reason this was done. Frequently it indicates an event of s
6. Serum glucose >150 mg/dl: Look for serum glucose values exceeding this lev
7. Hyperkalemia (High serum potassium): Look for lab values outside of these r
   following are the normal ranges for patients based on age: 0 – 3 months = 3.7
   3 months – 1 year = 4.1 – 5.3 mEq/L: 1 year – adult = 3.6 – 5.0 mEq/L
8. Look in the progress notes for documentation; includes cardiac arrest, respira
   and respiratory distress; patients in ICU requiring emergency intubation. This
